# Bioactive Antioxidant Compounds from Chestnut Peels through Semi-Industrial Subcritical Water Extraction

**DOI:** 10.3390/antiox11050988

**Published:** 2022-05-18

**Authors:** Christian Cravotto, Giorgio Grillo, Arianna Binello, Lorenzo Gallina, Mariló Olivares-Vicente, María Herranz-López, Vicente Micol, Enrique Barrajón-Catalán, Giancarlo Cravotto

**Affiliations:** 1 GREEN Extraction Team, INRAE, UMR 408, Avignon University, 84000 Avignon, France; christian.cravotto@alumni.univ-avignon.fr; 2 Department of Drug Science and Technology, University of Turin, Via P. Giuria 9, 10125 Turin, Italy; giorgio.grillo@unito.it (G.G.); arianna.binello@unito.it (A.B.); lorenzo.gallina@edu.unito.it (L.G.); 3 Instituto de Investigación, Desarrollo e Innovación en Biotecnología Sanitaria de Elche (IDiBE), Universidad Miguel Hernandez (UMH), 03202 Elche, Spain; maria.olivaresv@umh.es (M.O.-V.); mherranz@umh.es (M.H.-L.); vmicol@umh.es (V.M.); 4 CIBEROBN (Physiopathology of Obesity and Nutrition CB12/03/30038) Carlos III Health Institute, 28029 Madrid, Spain

**Keywords:** subcritical water extraction, chestnut peels, polyphenols, tannins, semi-industrial scale, bioactivity, adipocytes, ultra-nano filtration

## Abstract

Chestnut peels are a poorly characterized, underexploited by-product of the agri-food industry. This raw material is rich in bioactive compounds, primarily polyphenols and tannins, that can be extracted using different green technologies. Scaling up the process for industrial production is a fundamental step for the valorization of the extract. In this study, subcritical water extraction was investigated to maximize the extraction yield and polyphenol content. Lab-scale procedures have been scaled up to the semi-industrial level as well as the downstream processes, namely, concentration and spray drying. The extract antioxidant capacity was tested using in vitro and cellular assays as well as a preliminary evaluation of its antiadipogenic activity. The temperature, extraction time, and water/solid ratio were optimized, and the extract obtained under these conditions displayed a strong antioxidant capacity both in in vitro and cellular tests. Encouraging data on the adipocyte model showed the influence of chestnut extracts on adipocyte maturation and the consequent potential antiadipogenic activity. Chestnut peel extracts characterized by strong antioxidant power and potential antiadipogenic activity were efficiently obtained by removing organic solvents. These results prompted further studies on fraction enrichment by ultra- and nanofiltration. The semi-industrial eco-friendly extraction process and downstream benefits reported here may open the door to production and commercialization.

## 1. Introduction

Biowaste from the forest and agro-food industries can be considered a valuable bioactive compounds resource that has several applications in the food, pharmaceutical, and cosmetic industries. It follows that, during recent years, we have witnessed its reuse to obtain high added-value materials, with the further purpose of saving costs and translating recycling into a benefit for the environment. Notably, according to the Sustainable Development Goals defined by the EU, there is an aim to halve food waste per capita by 2030 [1].

The chestnut (*Castanea sativa* Mill.) is a deciduous tree belonging to the angiosperm family *Fagaceae* that can reach 30–35 m in height and can live up to 1000 years. Its distribution area ranges from southern Europe and North Africa to northwestern Europe and eastward to western Asia. Regarding Europe, chestnuts cover an area of more than 2.5 million ha, most of which (89%) is concentrated in France and Italy, followed by Spain, Portugal, and Switzerland. In 2019, the FAO (Food and Agriculture Organization of the United Nations) reported that 582,545 ha and 2,321,780 tons correspond to the global harvested area and chestnut production, respectively [2]. Moreover, in Europe, traditional orchards for fruit production (which are primarily roasted, candied, boiled, dried, or transformed into gluten-free flour) cover approximately 20% of chestnut forests [3], and the processing chain (e.g., peeling) necessary for most related employment leads to the generation of waste matter. The integument, which represents an important part of this residual material, is composed of a hard hull (outer shell) and inner thin skin (inner shell), constituting 1.5–8.9% and 6.3–10.1%, respectively, of the whole fresh fruit weight.

Commonly, the chestnut peeling process can be done through two different techniques, one known as “*brulage*” and the other that involves a water steam treatment, both giving an inner and outer shells mixture as residue [4]. Although still rich in bioactive compounds (e.g., antioxidants), this chestnut by-product is commonly discarded or burned as fuel, and despite the growing interest in this type of raw material, to the best of our knowledge, much work needs to be carried out to valorize it [5,6,7].

Polyphenols are among the more interesting secondary metabolites produced by the vegetal kingdom. These bioactive compounds have well-established activities, the first of which is that they are reactive oxygen species scavengers that can contribute to human health, especially with regard to the prevention of degenerative disease [8,9,10]. Moreover, obtaining antioxidant compounds from natural sources, such as vegetal matter, is important for the replacement of synthetic compounds, the safety of which is often questioned [11].

Generally, the extraction protocol constitutes the foremost step in bioactive compound recovery and often involves organic solvents alone or in combination. A crucial role is therefore played by the overall operations of this critical procedure. Notably, although high extraction yields are important in terms of process viability, values such as the total phenolic content (TPC), bioactive compound profile, and antioxidant activity of the obtained products must be taken into account. In fact, despite often leading to higher yields, prolonged extraction times and high temperatures can promote phenolic compound oxidation, thus decreasing the antioxidant properties shown by extracts [12,13]. For that reason, bioassay-guided screening remains the basic procedure for characterizing new natural products with defined biological activity [14,15]. It must also be considered that although obtaining pure natural bioactive compounds remains a topic of interest for researchers, alternative approaches are focused on the synergic activity of secondary plant metabolite blends and on the enriched fraction of the extracts [16].

Several studies on chestnut industry wastes have demonstrated that they can be a potential source of bioactive compounds [17,18]. Phenolic compounds in outer and inner chestnut shells reach amounts within the range of 2.7–5.2% (*w/w*), while approximately 36% (*w/w*) is made up of polysaccharides [19], and a water content of approximately 20% has been reported for these by-products [17]. The extraction procedure selection can heavily influence the obtained TPC values, and the main subdivision of these types of secondary metabolites is commonly between simple phenolic acids, flavonoids, and tannins (condensed and hydrolyzable). In particular, gallic acid (the main phenolic representative of hydrolyzable tannins), protocatechuic acid, chlorogenic acid, epicatechin, syringic acid, ellagic acid, *p*-coumaric acid, sinapic acid, ferulic acid, and scopoletin are among the identified compounds [20,21,22]. Generally, alcoholic or hydroalcoholic mixtures are more efficient than acetone in obtaining low molecular weight polyphenols, while higher yields of high molecular weight flavonoids can be reached with aqueous acetone [7,23]. As mentioned above, heating can increase the extraction of these compounds, but structural degradation can also occur [24], especially for small molecules, such as gallic acid and protocatechuic acid, which can be more sensitive to high temperatures than flavonoids or tannins. Moreover, pyrogallol and protocatechuic acid can originate from the thermal degradation of tannins [25].

In accordance with ecological, economic, and innovative chemistry concepts, green extraction procedures are currently becoming mandatory to obtain added-value natural products from vegetal material and agro-food wastes. Compared to conventional procedures, the environmental impact of the whole extraction process can be reduced by limiting the organic solvent, saving energy consumption, and reducing the extraction time, number of operations, and waste generation while simultaneously enhancing the extraction process efficiency and the quality of the obtained product [26]. In recent years, this trend in the treatment of chestnut processing waste has been demonstrated by the growing number of studies directed toward the use of hot/boiling water as a solvent [17,21,27], and the inner shell aqueous extract has been referred as the richer in total polyphenols, ortho-diphenols, tannins, and flavonoids when compared to leaves, burs, and outer shells [28]. Moreover, hydrothermal treatment has been described as an environmentally friendly technology suitable for recovering high value-added compounds (oligosaccharides and antioxidants) from chestnut shells [29], and alkali solutions have been tested in polyphenols and hydrolyzable/condensed tannins obtained from chestnut peels [27]. Interestingly, a membrane technology has been used to concentrate the phenolic fraction present in the liquor derived from the hot alkaline treatment of chestnut husk by 15% [30]. Unconventional green techniques such as ultrasound-assisted extraction (UAE) [31,32], microwave-assisted extraction (MAE) [33], and supercritical fluid extraction (SFE-CO_2_) [34] have also been studied in chestnut shell recycling.

Recently, subcritical water extraction (SWE) has been successfully applied for the recovery of value-added compounds such as antioxidants (phenols and flavonoids) from various vegetal materials [35,36], and it represents an eco-friendly technique suitable for selective extraction procedures, viable for agro-food waste treatment, and attractive for industrial processing to make safe and high-quality products [37]. Over a temperature range between 100 °C and the critical temperature of 374 °C (commonly from 100 to 250 °C), water is liquid under sufficient pressure (normally 10 to 100 bar) and is called subcritical, near-critical, or pressurized hot water. Under these conditions, water has many advantages regarding extraction efficiency and selectivity and can be used to replace organic solvents such as methanol. The most important effect of the water temperature increase is undoubtedly the weakening of hydrogen bonds, resulting in a lower dielectric constant (from 80 ε to approximately 30 ε), with values that fall between those of methanol (33 ε) and ethanol (24 ε) that allow for efficient extractions of moderately polar and non-polar target compounds [38]. Even if studies about the large-scale operation and the design of industrial equipment remain to be deepened, it is peculiar that the dielectric constant can be varied as a function of the temperature and applied pressure [39]. Moreover, the ionic constant increases with temperature increases to 300 °C, beyond which it decreases again; a resulting lower density, viscosity, and surface tension together with a higher diffusion coefficient are due to the higher kinetic energy and mobility of water molecules under subcritical conditions [40]. All these characteristics have ensured that the applications of this promising technology are constantly growing in multiple extraction fields [41]. Notably, SWE conditions can also be reached through a hybrid technique involving rapid microwave (MW) heating under controlled temperature and pressure sealed vessels in which water is heated above its boiling point [42].

When SWE target compounds possess structures similar to those of phenolic compounds, they can be totally or partially degraded as a consequence of the applied temperature and pressure, thus generating different molecules, such as gallic acid and catechin, from which pyrogallol and protocatechuic acid can originate, respectively. Attention must be given when further processing steps such as extract drying are performed because of physical factors (e.g., light and heat) that can also lead to bioactive compound degradation.

Although Pinto et al. [25] have very recently reported powdered shell extraction with subcritical water monitoring TPC values, antioxidant and antimicrobial activities, phenolic profiles, and evaluations of the extract safety for cosmetic purposes through in vitro assays [43], as far as we know, polyphenol extraction from chestnut shells via SWE has been poorly investigated to date.

The aim of this work was to design a semi-industrial green protocol to obtain a bioactive extract starting from chestnut peel (a blend of inner and outer chestnut shells) processing and employing SWE as an eco-friendly procedure. To overcome the problem related to removing large quantities of water from the obtained extracts, the potential application of different sample concentrations and enrichment systems, such as ultrafiltration and membrane filtration, was evaluated. The antioxidant capacity of the obtained products was evaluated first using a collection of in vitro antioxidant tests and then in a cellular model composed of adipocytes in which the antiadipogenic activity was also preliminarily tested.

## 2. Materials and Methods

### 2.1. Chemicals

Reagents for colorimetric assays (Folin–Ciocalteu, DPPH, pyrocatechol violet and cinchonine hemisulfate, Trolox^®^, EDTA, sodium carbonate, sodium acetate, and copper sulfate) were purchased from Sigma-Aldrich (St. Louis, MO, USA). Acetonitrile CHROMASOLV^®^ (gradient grade, for HPLC, ≥99.9%) for LC-MS analysis was purchased from Sigma-Aldrich, and Milli-Q H_2_O was obtained in the laboratory using a Milli-Q Reference A+System (Merck Millipore, Burlington, MA, USA). Standards for LC-MS (gallic acid, catechin, epicatechin, ellagic acid, and (epi) gallocatechin) were purchased from Sigma-Aldrich.

### 2.2. Chestnut Peel Matrix

Chestnut peels were kindly provided by Castellino SRL (Villanova Mondovì, Italy). This biomass was stored at room temperature (RT) in a dry environment to avoid metabolite degradation and used as is.

### 2.3. Microwave-Assisted Subcritical Water Extraction (MASWE)

Chestnut peels (20 g) were mixed with the desired amount of water at a 1:20 or 1:30 solid/liquid (S/L) ratio (400 or 600 mL, respectively). The mixture was left to moisturize for 5 min in a 1 L Teflon line, which was then introduced into an MW multimodal reactor (SynthWAVE, Milestone, Bergamo, Italy) able to exploit an external inert gas feeding (N_2_). For each test, an appropriate purging with N_2_ was performed three times to remove oxygen traces from the system, reducing oxidative stress on the biomass. The reaction chamber was then pressurized with the necessary amount of N_2_ to avoid water ebullition (5–25 bars). The samples were heated at different temperatures (100, 120, 150, and 220 °C) with a maximum irradiation power of 1500 W. The temperature was maintained for the desired amount of time (0, 2.5, 5, 10, 15, 20, 30, and 45 min under magnetic stirring at 650 rpm). The resulting solution was filtered under a vacuum, while thoroughly washing the biomass with fresh water. The dry extract was recovered by freeze-drying (LyoQuest-85, Telstar, Madrid, Spain), weighed, and stored at 4 °C for further analyses. Each extraction was performed in triplicate to validate the reproducibility of the experimental results and the percentage standard deviation was consequently calculated and reported as error bars in graphs.

### 2.4. Colorimetric Assays

#### 2.4.1. Total Phenolic Content (TPC)–Folin-Ciocalteu Assay

The TPC was determined using the Folin–Ciocalteu assay, as previously described [44]. In brief, polyphenol quantification was performed by applying a calibration curve of gallic acid as a reference compound (with dilutions between 5 and 250 µg/mL) in aqueous solutions. Dried extracts were dissolved in deionized water at concentrations of 4–6 mg/mL. The gallic acid and sample solutions (250 µL) were dispensed into borosilicate test tubes. The following solutions were added sequentially for each test: 500 mL of Na_2_CO_3_ solution (10% *w/v*), 4 mL of distilled water, and 250 µL of Folin–Ciocalteu reagent (diluted 1:1 with distilled H_2_O). The resulting mixtures were vigorously shaken and kept at room temperature for 25 min before the analysis. A Cary 60 UV-Vis spectrophotometer (Agilent Technologies, Santa Clara, CA, USA) was used to read the absorption at 740 nm in a quartz cuvette (1 cm). The TPC was expressed as mg/g of gallic acid equivalents (GAE) over the extract and over the dried matrix. The measurements were performed in triplicate.

#### 2.4.2. Tannin Determination, Cinchonine Hemisulfate Assay

Tannin quantification was performed by adapting Peri and Pompei [45]. Specifically, 0.6 mL of extract was mixed with 0.6 mL of cinchonine hemisulfate solution (0.5% *w/v*) in a 1.5 mL centrifugal tube. The mixture was left overnight at 4 °C to facilitate precipitation. The supernatant fraction was then recovered after 5 min of centrifugation at 26,000 rpm. The precipitate represents the tannic fraction. The Folin–Ciocalteu assay was conducted on the supernatant, and the total tannins were expressed as GAE equivalents by difference. Hydrolyzable and nonhydrolyzable tannins were determined using an adaptation of the method by Scalbert et al. [46]. The precipitate obtained in the first step was resuspended in 0.6 mL of EtOH_aq._ (1:1). Then, 0.5 mL of this solution was mixed with 0.25 mL of H_2_O/HCl 36% (5:2 *v/v*) and 0.25 mL of formaldehyde 4.8%. The mixture was incubated overnight and then centrifuged for 5 min at 26,000 rpm. The supernatant contains the hydrolyzable fraction, as determined by the Folin–Ciocalteu assay. The nonhydrolyzable tannins were determined by taking the difference between the total and hydrolyzable tannins.

#### 2.4.3. Antioxidant Activity, DPPH∙Assay

The radical scavenging activity of the extracts was evaluated using the stable free radical DPPH∙ according to the method described by Brand-Williams et al. [47]. The details of the procedure and calculations have already been reported in a previous study [48]. The bleaching rate of the DPPH∙ radical was monitored in the presence of chestnut extracts and in a Trolox^®^ (antioxidant standard) solution, for the sake of comparison, to evaluate the IC50 (the half-maximal concentration of extract necessary to halve the initial concentration of DPPH∙ at equilibrium). Various concentrations of extracts were analyzed at 515 nm (Cary 60 UV-Vis spectrophotometer, Agilent Technologies, Santa Clara, CA, USA). The collected data were processed with Bobo Least Squares software (ver. 0.9.1.) [49] to establish an accurate probit regression. Blank samples containing the extracts without the DPPH∙ reagent were adopted to evaluate and subtract the matrix effect and avoid interference at the analytical wavelength.

The radical scavenging activity was expressed as mg of compound/dried extract per mL solution. The Trolox^®^ equivalents mmol/g of the extract were calculated according to the IC_50_ values (3.94 µg of Trolox^®^/mL, corresponding to 0.0157 µmol/L).

#### 2.4.4. Cu Chelating Activity, Pyrocatechol Assay

The ability of the chestnut peel extract to chelate Cu^2+^ was assessed using the pyrocatalyst violet (PV) assay, as previously reported [50].

PV binds the Cu^2+^ not bound by polyphenols in the slightly acidic medium, forming a dark red colored complex. In the presence of a chelating agent, the color shifts to pale yellow, thus allowing for the estimation of chelating activity using the rate of color reduction. A total of 600 µL of a water solution of chestnut peels (extract or formulated product) was mixed with 4 mL of sodium acetate buffer (4.10 g/L, pH 6.0). Then, 600 µL of copper sulfate (50 mg/L) solution was added, and the mixture was left to react for 2 min. Lastly, 170 µL of a PV water solution (773 mg/L) was added. The mixture was stirred for 10 min and allowed to react for 10 more minutes without stirring, then the absorbance was read at 632 nm on a Cary 60 UV-Vis spectrophotometer (Agilent Technologies, Santa Clara, CA, USA).

The inhibition of PV–Cu^2+^ complex formation is expressed as the % of inhibition against a reference solution, which is prepared the same way as the samples but contains water in place of chestnut peel extract, and the inhibition is calculated using Equation (1):

(1) $$\%\;\mathrm{of}\;\mathrm{inhibition}=\frac{\mathrm{ABSref}-\mathrm{ABSsample}}{\mathrm{ABSref}}$$

The percent inhibition was calculated for various concentrations of each sample, and the IC_50_ was calculated by probit regression (collected data were processed by Bobo Least Squares software (ver. 0.9.1.) [49].

The sample IC_50_ values were then compared with the IC_50_ values obtained from an EDTA solution to express the results as EDTA equivalents.

### 2.5. Kinetic Model

The hyperbolic model by Peleg (see Equation (2)) was applied to evaluate the extraction kinetics and to determine the point of maximum extraction rate by means of the related constants. The method was applied according to a previous study [51]. In brief,

(2) $$C(t)=C_{0}+\frac{t}{k_{1}+k_{2}t}$$

*C(t)* is the concentration of the extract after extraction time *t*, while at the beginning of the process, *C_0_* is equal to 0. The *Peleg initial extraction rate* (*k_1_*) is related to the starting extraction rate (B_0_) necessary to extrapolate the extraction rate at each moment of the process (*B_t_*). This parameter can be used to calculate the instant of maximum extraction speed, which is crucial for an industrial transposition of the process. The *Peleg capacity constant* (*k_2_*) is related to the highest extraction yield at the steady state (*C_max_*). Equation (2) can be conveniently linearized, providing a fast and easy way to extrapolate k_1_ and k_2_ as the intercept and slope, respectively, as defined by the linear interpolation of experimental values.

The obtained hyperbolic curve describes a time-dependent extraction trend. This model is useful for displaying the horizontal asymptote of *C_max_* and the extraction rates (slope of the curve). Furthermore, the knee point can be exploited to determine the best trade-off between productivity and process extent.

### 2.6. LC-MS Analysis and IR Spectra

The LC-MS analysis was performed on a Waters FractionLink equipped with a diode array detector (DAD) and a mass spectrometer (single quadrupole) supported by MassLynx V4.1 software. MS detection was performed in ESI+ mode (capillary: 3.00 kV; cone: 20.00 V; source temperature: 110 °C; and desolvation temperature: 220 °C, mass span 100–800). The DAD was monitored at 280 and 335 nm. Chromatographic separation was performed with a Waters Xbridge column (C18 4.6×150mm, 5 µm), eluents: A = Water/TFA 0.1%, and B = I/TFA 0.1%; gradient (min, %B): 0, 5; 9.98, 5; 42.38, 15; 57.34, and 30; 69.80, 60; 76.29, 100; 88.75, and 100. Flow: 1 mL/min; injection volume: 20 µL.

IR spectra were recorded using a Spectrum Two ATR (Perkin Elmer, Waltham, MA, United States). Ten milligrams of each sample was analyzed, and data were collected from 500 to 4000 cm^−1^ over 16 scans in transmittance mode.

### 2.7. Membrane Filtration, Ultra and Nanofiltration

A lab-scale membrane filtration skid (PB100, Hydro Air research Srl, Lodi, Italy) with a 3 L tank was applied for the membrane filtration of the chestnut peel extract solution prior to clarification by means of vacuum filtration. The system was equipped with a DKU 1812 (150–300 Da, 0.38 m^2^ filtering area) for nanofiltration (NF) and an SDR5-1812 (5000 Da, 0.33 m^2^ filtering area) for ultrafiltration (UF). Approx. 1 L of solution was processed at a constant flow rate of 370 L/h, imposing a suitable counterpressure (4 bar for NF and 2 bar for UF, maintained across the entire process). The retentate stream was continuously recirculated to the feeding tank, while the permeate was collected in a graduated cylinder to monitor the filtration trend. The process was performed up to the incipient precipitation of the extract. Both fractions were recovered and freeze-dried for further analysis.

### 2.8. Semi-Industrial Scale Subcritical Water Extraction (SWE)

Semi-industrial scale Subcritical Water Extraction (SWE) was performed in a customized pre-industrial scale reactor (Tropical Food Machinery SRL, Busseto, Italy).

The system is composed of a 40 L recirculation tank providing a hot water reservoir at approx. 90 °C). Water passes through a heat exchanger (fed by steam), heating to the desired temperature in a closed loop. The flow is then redirected to two extraction tanks (65 L stainless steel vessels), preventing cold water contact with the matrix and the consequent slow heating, which leads to an extended extraction time and undesired gradients.

The overall reactor volume amounts to 180 L, comprising 10 L of dead volume from piping. For the chestnut peel extraction, 30 kg of matrix was loaded into each extraction tank for a total of 60 kg. The tanks were sealed, and the extraction was performed for 30 min at 150 °C. The extract samples were then dried and analyzed to determine the extraction yield and TPC. Extraction was performed in triplicate to validate the reproducibility of the experimental results and the percentage standard deviation was consequently calculated and reported as error bars in graphs.

### 2.9. Biological Activity

#### 2.9.1. Chemicals and Reagents

Dulbecco’s Modified Eagle’s Medium (DMEM), calf and fetal bovine serum, and a mixture of penicillin/streptomycin were purchased from Thermo Fisher Scientific (Waltham, MA, USA). 3-Isobutyl-1-methylxanthine (IBMX), dexamethasone, insulin, dimethyl sulfoxide (DMSO), and phosphate-buffered saline (PBS) were provided by Sigma-Aldrich (St. Louis., MO, USA).

#### 2.9.2. Differentiation of 3T3-L1 Preadipocytes into Mature Adipocytes

The pre-adipose cell line 3T3-L1 (ATCC^®^ CL-173TM) was cultured in DMEM supplemented with 10% bovine calf serum, 100 U/mL penicillin, and 100 μg/mL streptomycin under optimal conditions (37 °C, 95% humidity, and 5% CO_2_). To obtain mature adipocytes, confluent preadipocytes were induced with DMEM containing high glucose (25 mM), 10% fetal bovine serum, 0.5 mM IBMX, 1 μM dexamethasone, and 10 μg/mL insulin for 48 h, followed by high glucose DMEM supplemented with 10% fetal bovine serum and 10 μg/mL insulin for 7 days as described in [52].

#### 2.9.3. Quantification of Reactive Oxygen Species (ROS) Levels and Triglyceride Accumulation

**Figure 1:**
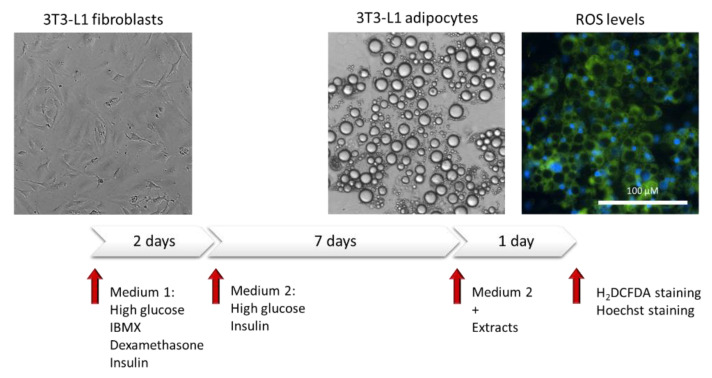
Cellular treatment scheme for ROS quantification. Representative images are shown above the timeline.

High glucose-induced adipocytes have been used as a cell model in studying oxidative stress. Adipocytes were treated with the extracts at several concentrations for 24 h. Depending on their solubility, the extracts were previously dissolved in DMSO and reconstituted in culture medium for sterilization. The amount of DMSO used in the culture did not exceed 0.5%. The intracellular ROS levels were determined by using the fluorogenic dye 2′,7′-dichlorodihydrofluorescein diacetate (H_2_DCFDA, Sigma-Aldrich). In brief, adipocytes treated with the extracts for 24 h were incubated with H_2_DCFDA at 30 μM for 30 min at 37 °C. Then, the cells were washed with PBS, and the fluorescence was measured at 495 nm excitation and 529 nm emission wavelengths using a cell imaging multimode microplate reader (Cytation 3, Biotek Instruments, Winooski, VT, USA). Fluorescent images were obtained using the same equipment. In addition, the cytotoxicity of the extracts was eliminated by staining the nuclei with Hoechst 33,342 dye (Invitrogen, Thermo Fisher Scientific). A diagram of the process is shown in Figure 1.

The lipid content of the adipocytes was assessed using the AdipoRed™ reagent (Sigma-Aldrich (St. Louis., MO, USA). In brief, the supernatant was removed from the cells, and the cells were washed carefully with PBS. Then, AdipoRed™ was added, and the cells were incubated for 15 min at room temperature. Triglyceride accumulation was measured using a microplate reader at 485 nm excitation and 572 nm emission (Cytation 3, Biotek Instruments, Winooski, VT, USA). Fluorescent images were obtained using the same equipment. Each test was performed in triplicate to validate the reproducibility of the experimental results and the percentage standard deviation was consequently calculated and reported as error bars in graphs.

## 3. Results and Discussion

### 3.1. Microwave-Assisted Subcritical Water Extraction (MASWE)

#### 3.1.1. Screening

A first extraction screening was performed at different temperatures, fixing 30 min as the process time. Several conditions were tested to investigate the role of subcritical water, even considering two different S/L ratios, by evaluating the mass transport enhancement.

**Figure 2:**
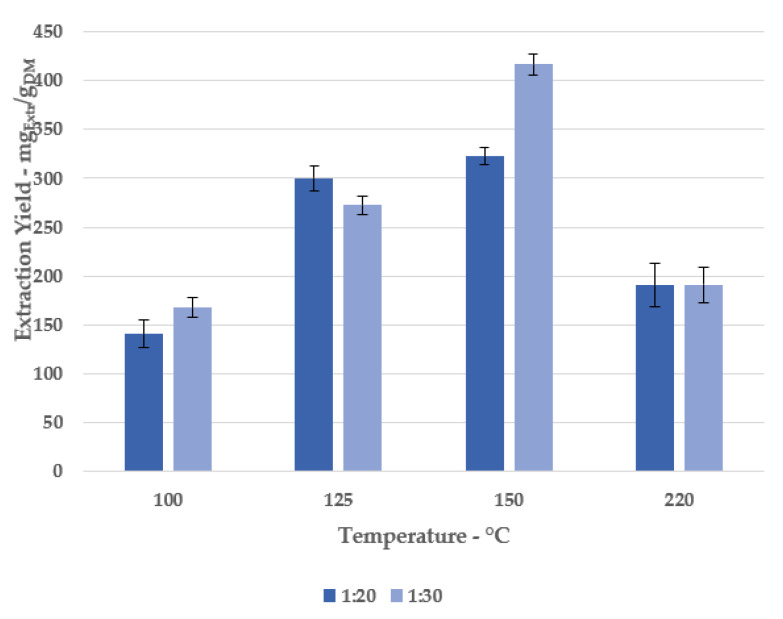
Extraction yields at different solid/liquid ratios and temperatures.

The extraction yield (as expressed as dry matter, DM) was adopted as the main parameter to describe the extraction trend (see Figure 2).

It is possible to note an increasing amount of product, proportional to the temperature, dropping at 220 °C. Concurrently, negligible variations can be reported for the 1:20 and 1:30 S/L ratios, with the exception of 150 °C (322.37 mg_Extr_/g_DM_ vs. 416.56 mg_Extr_/g_DM_, respectively).

**Figure 3:**
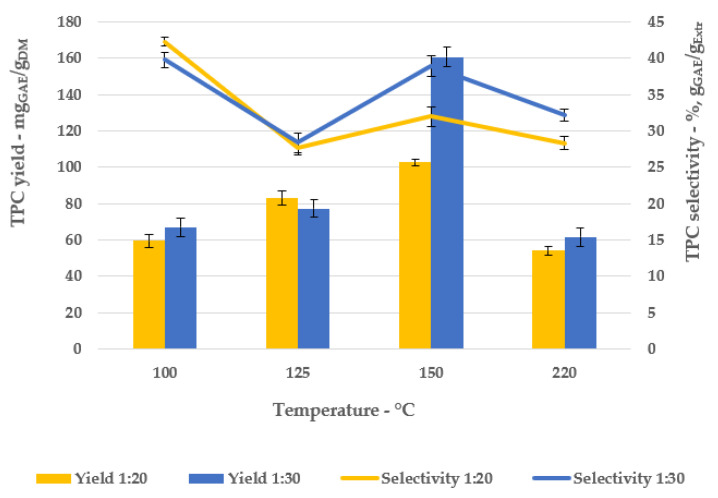
TPC, yield, and selectivity.

Another key parameter adopted to describe the SWE process is the TPC evaluation, which was expressed both as yield (mg_GAE_/g_DM_) and selectivity (%, g_GAE_/g_Extr_), as reported in Figure 3.

Similar to the dry extraction yield, the TPC trends (yield and selectivity) confirm the temperature proportionality, excluding the test performed at 220 °C, achieving the best SWE protocol at 150 °C, particularly for the 1:30 S/L ratio (160.70 mg_GAE_/g_DM_ and 8.96%, g_GAE_/g_Extr_).

#### 3.1.2. Temperature-Related Degradation

The results observed for the 220 °C extraction suggested the occurrence of temperature-related degradation. This outcome diverges from previous studies on chestnut peels, in which the best extraction yields were achieved at 220 °C [26]. To clarify this divergence, a deeper study on the influence of high temperatures on polyphenol degradation was performed. The degradation was assessed using a simple test: an aliquot of extract recovered under the optimized conditions (150 °C, 30 min) was suspended in water with the same S/L ratios and re-subjected to MW irradiation at 220 °C. The results were monitored according to the dry yield and TPC evaluations, with the percent degradation being reported in Table 1. The concept of “degradation” here is intended as a sum of condensation phenomena (leading to insoluble compounds lost during work-up procedures) [53], together with transformations of metabolites, such as the decarboxylation of gallic acid from tannins in pyrogallol or catechin to 3,4-dihydroxybenzaldehyde and protocatechuic acid [54,55].

From Table 1, it is possible to observe a large contraction in dry extract recovery (approx. 70% for both S/L ratios), supporting the formation of condensation products in accordance with the literature (high molecular weight and humin-like compounds). Thus, the quantity of soluble metabolites declines, concurrently reducing the available polyphenolic content and increasing the solid residues removed by filtration at the end of the process. Additionally, the overall TPC values experienced a reduction, and it is worth noting the TPC selectivity abatement, which provides evidence of the extract quality.

Furthermore, the LC-MS analysis reported a radical transformation in chromatograms registered for extracts subjected to 220 °C treatment. Interestingly, the chromatographic patterns were matched for the extracts and re-irradiated samples. The same statement does not apply to the optimized extraction protocols at 150 °C, in which tannin signals (approx. R_t_ of 50–70 min) are clearly visible, in contrast with the re-irradiated sample (Figure S1a vs. Figure S1b). Further investigations reveal the occurrence of pyrogallol (molecular weight 126.11 g/mol) after the 220 °C treatment, which is otherwise not detectable for the 150 °C sample (see Figure S2). This evidence fits the assumptions made thus far, with the rise of this compound from the thermal degradation (T > 150 °C) of hydrolyzable tannins being well known in the literature [38].

#### 3.1.3. Kinetic and Time-Related Degradation

**Figure 4:**
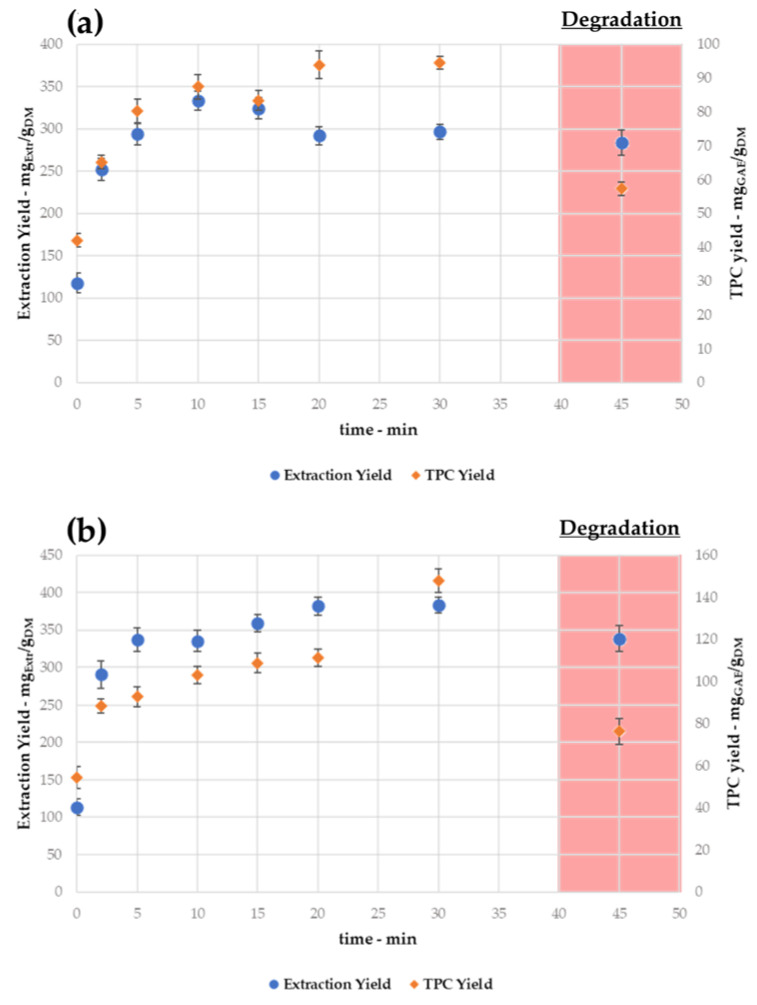
SWE kinetics at 150 °C for different S/L ratios. (**a**) 1:20; (**b**) 1:30.

Once the optimized extraction temperature was defined at 150 °C, the SWE kinetics were investigated, depending on the dry and TPC yields, at 1:20 and 1:30 S/L ratios (see Figure 4a,b). For the sake of comparison, the sample recovered at time “0” was obtained only by means of the heating ramp (which was constant across all the tests).

As already stated in Section 3.1.1, 1:30 appears to be the best performing S/L ratio according to the extraction trends. In addition, it is worth noting that for both S/L ratios, the productivity and quality of samples at 45 min show a general contraction. Hence, once more, the overall yields (both dry and TPC) suggest a degradation episode, which is time-dependent, in contrast with Section 3.1.2.

To investigate this phenomenon, an extract produced at 30 min (150 °C) was recovered from the biomass, resuspended, and heated at 150 °C for a total of 45 min. The product was characterized as described in Section 3.1.2 for the degradation percentage, with one for every S/L ratio (see Table 2).

The degradation trend appears to be less extended compared to the temperature-related degradation (pprox. 17–18% vs. 70–72% dry yield loss), with comparable TPC variations. Thus, as expected, thermal stress has harsher effects on metabolite stability, while protracted treatments start to exhibit milder decay only after 30 min.

**Figure 5:**
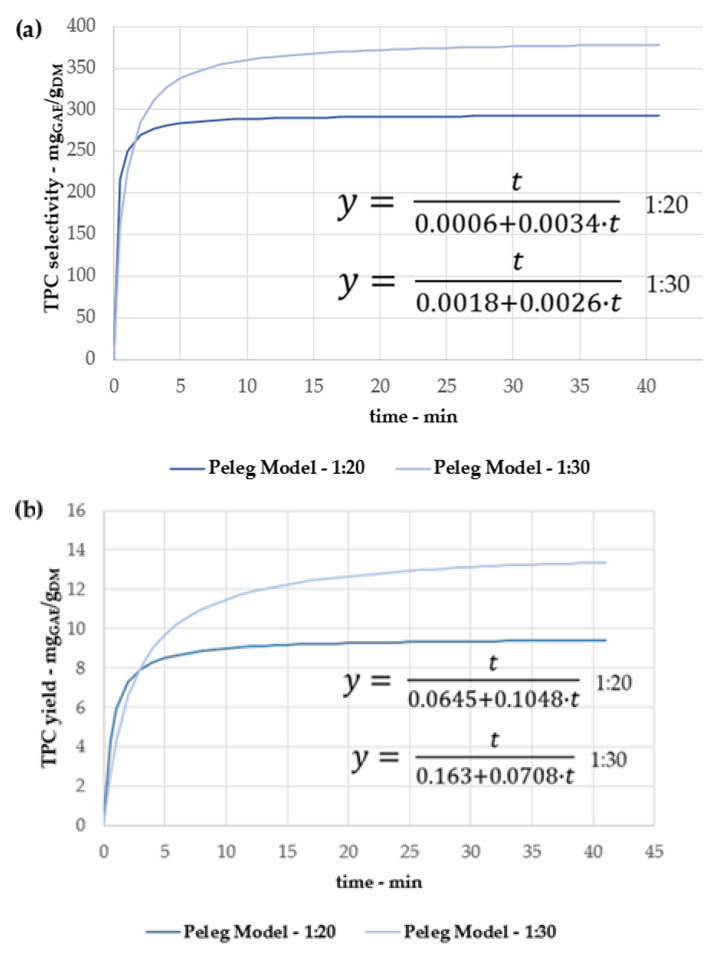
Peleg model for 1:20 and 1:30 S/L ratios, chestnut peel extractions at 150 °C. (**a**) TPC selectivity; (**b**) TPC yield.

When removing the extraction outcomes re-conducted to degradation, the TPC yields and selectivities (Figure 4a,b) were exploited to describe the kinetic processes using the Peleg model. The interpolation was generally high (R^2^ > 0.95), demonstrating a good fit with the experimental values (see Figure 5a,b).

The extrapolations consolidate the conclusion that the protocol addressing a 1:30 consistency has the best outcomes (both for extraction and TPC yields) after 30 min, while the 1:20 process stably reaches lower values after 20 min. Working with a 1:20 mixture led to an extraction yield contraction of ~22%, reaching a decrease of ~30% for the TPC yield.

**Figure 6:**
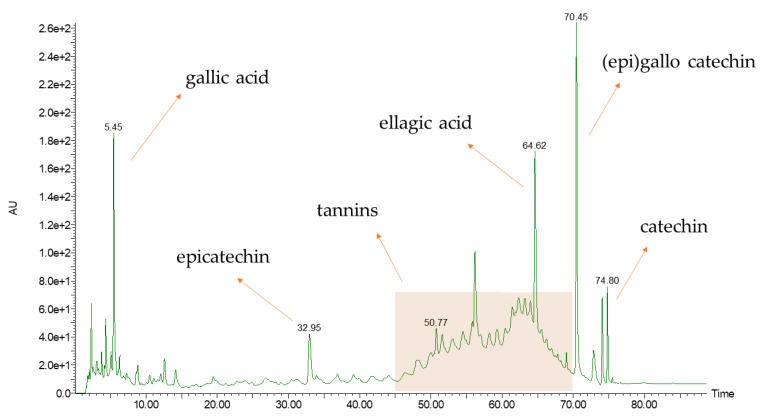
LC-MS of chestnut peel extract, DAD chromatograms of extract at 150 °C, 30 min.

From the Peleg equations (see Figure 6), it is possible to extrapolate B_0_ and *C_max_* as kinetic parameters, which is useful to describe the process extensively and to validate the conclusions concerning the optimized protocol (see Table 3). Even if the extraction rates are higher for 1:20 (resulting in an overall faster extraction), the determined maximum achievable concentration (*C_max_*) indicates the 1:30 procedure is the most promising one, and thus it was selected as the optimized condition for further experiments.

#### 3.1.4. Chestnut Peel Optimized Extract Characterization, LC-MS, Tannin Quantification and Antioxidant Activity

The extract of chestnut peels obtained under optimized SWE conditions was characterized to describe its composition by the main metabolites. First, LC-MS screening helped to identify and quantify the five main polyphenolic compounds (Figure 6, Table 4) (by means of external standards). ESI + scans are reported in the supporting information (Figures S3–S7).

The LC-MS analysis confirmed the expected main components [26], with a predominance of (epi)catechin and (epi)gallocatechin (112.85 and 30.20 mg/g_EXTR_, respectively). Moreover, the tannin region (approx. R_t_ of 50–70 min) appears populated and requires further investigation. Thus, with an adapted cinchonine hemisulfate protocol (see Section 2.4.2), it was possible to determine the total tannins and their composition in hydrolyzable and nonhydrolyzable tannins (Table 5).

The reported results show how the main activity of the TPC assay actually depends on the high concentration of tannins, becoming effectively the main component of the chestnut peel SWE extract (82.84%, 76.49% of which are nonhydrolyzable).

Lastly, the antioxidant and Cu chelating activities of the optimized MASWE extract were determined using DPPH∙ and pyrocatechol assays, respectively. As reported in Table 6, the recovered product shows interesting antioxidant and chelating activities with 5.43 and 85.07 mmol/g_EXTR_ of Trolox^®^ and EDTA equivalents, respectively. The interesting chelating activity reflects the high tannin content of the extract, which is well known in the literature for its chelating properties.

### 3.2. SWE Semi-Industrial Extraction

Considering the volumes of chestnut peels available together with the required production volume of nutraceuticals and food supplements, it is necessary to evaluate the feasibility of scaled-up SWE. In this study, we tested a semi-industrial plant developed by C2FUT Srl (Busseto, PR, Italy), which is able to work with 40–50 kg divided into two pressurized extraction tanks. The biomass was charged in the reactor and continuously permeated by water (600 L/h) at 150 °C for 30 min. The semi-industrial scale product showed the same features as the lab-scale extract under MASWE, as reported in Table 7.

### 3.3. Spray-Drying and Formulations

To meet industrial needs, a spray-drying system (KD-SD-2000, Zhengzhou Keda Machinery and Instrument Equipment Co., Ltd., Zhengzhou, China) was tested to achieve a dry extract after the semi-industrial scale SWE. The protocol involved a 190–200 °C atomizer temperature, with 40% venting and a peristaltic pump speed of 20%. This approach allowed for the processing of high volumes of product streams, maintaining unaltered extract features with a non-expensive protocol. The conventional application of spray-dryers usually requires the addition of excipients, such as maltodextrins, crystalline cellulose, or silica. These compounds help to reduce the overall stickiness and enhance the product recovery, while the main drawback is the reduction of metabolite concentrations and thus the activity.

During this study, we tested the common excipients (low glycemic index maltodextrin, Avicell^®^ as crystalline cellulose, and Syloid^®^ as silica), comparing the results with an extract processed without any addition. Specifically, 50% maltodextrin, Avicell^®^, and Syloid^®^ were added at W/W ratios to a sample of chestnut SWE extract. As expected, the inert excipients led to a decrease in TPC yield, proportional to the additions, in which the maximum deviation is represented by maltodextrins, with a reduction of approx. 8% in the TPC yield, which was comprehensively negligible. The test performed using the extract solution without any chemical, by contrast, allowed for the recovery of unaltered sample, maintaining the final product with minimal TPC alteration. The ATR values of the formulations are reported in the supporting information for clarity (Figure S8–S14).

### 3.4. Biological Activity in Cellular Models

#### Quantification of ROS Levels by H_2_DCFDA Staining

**Figure 7:**
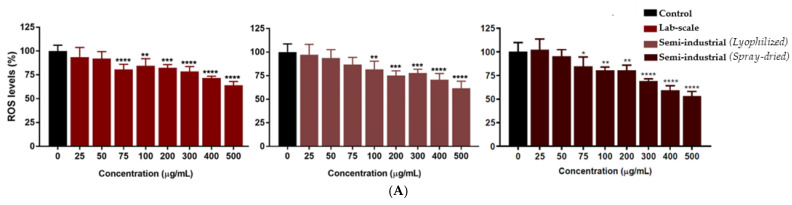

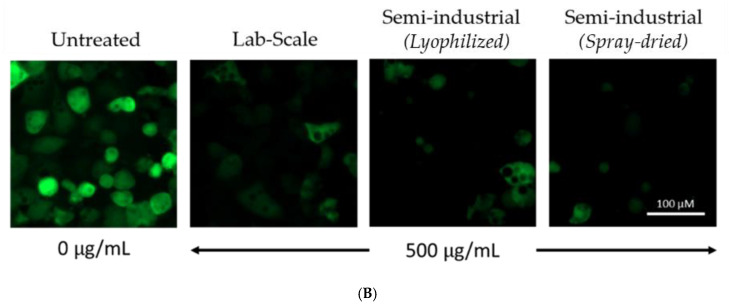
(**A**) ROS reduction levels in 3T3-L1 adipocytes (mean ± SD, *n* = 6). The untreated sample was considered 100%, and the reduction in the fluorescent signal was normalized against its value. All the values were viability-corrected as described in the methods section. Statistical significance was calculated using untreated samples (black bars) as a reference (* *p* < 0.05, ** *p* < 0.01, *** *p* < 0.001 and **** *p* < 0.0001). (**B**) Representative fluorescent images of H_2_DCFDA staining after treatment with 500 µg/mL extracts in comparison with the untreated sample.

Oxidative stress is one of the main signatures of metabolic imbalance in adipocytes, leading to metabolic disorders related to obesity and metabolic syndrome. To check the antioxidant capacity of the chestnut extract on this feature and to confirm the antioxidant capacity previously shown in the previous sections, adipocytes were treated with the different chestnut extracts as described in the Methods section at different concentrations (range: 25–500 µg/mL). All three extracts showed a significant reduction in ROS levels, as shown in Figure 7, with no loss of biological activity during either the upscaling process or the spray-drying process. This reduction is dose dependent in all cases and statistically significant from 75 µg/mL (100 µg/mL for the semi-industrial spray-dried sample). No toxicity was observed in any case (data not shown).

**Figure 8:**
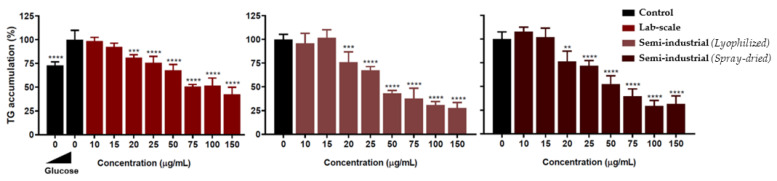
Triglyceride accumulation levels after AdipoRed™ staining. Undifferentiated control with low glucose is only shown in the left panel. Statistical significance was calculated using the high glucose-treated sample (full mature adipocytes) as a reference (* *p* < 0.05, ** *p*< 0.01, *** *p* < 0.001, and **** *p* < 0.0001).

**Figure 9:**
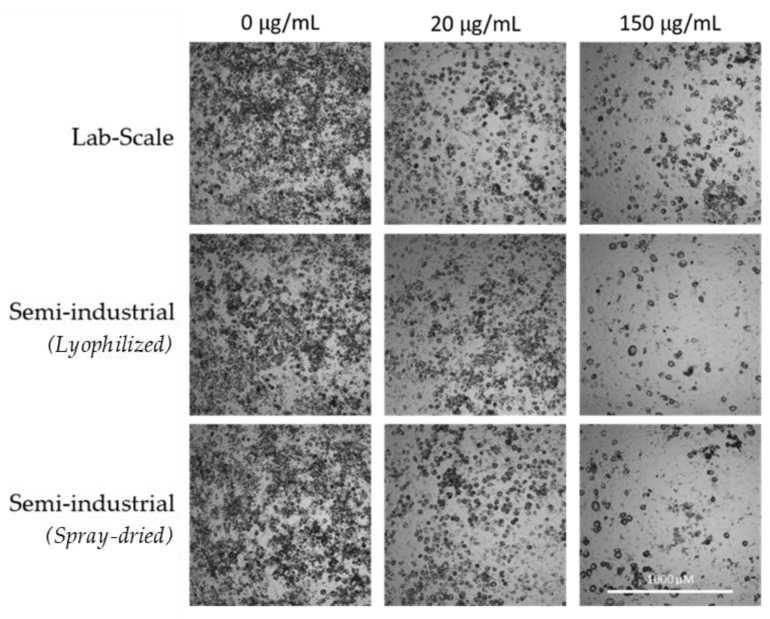
Representative microscope images of 3t3-L1 cells treated with different concentrations of the three tested extracts showing the accumulation of lipid droplets.

In addition to this antioxidant capacity in the adipocyte model, the influence of the chestnut extracts on adipocyte maturation was also preliminarily tested to identify potential antiadipogenic activity. In this way, different extract concentrations were added during the maturation process, from day 0 until day 9, when the triglyceride accumulation was measured as described in the Methods section. All the extracts were able to reduce triglyceride accumulation in a dose-response pattern and with statistical significance from 20 µg/mL (Figure 8). Once again, no influence of either the upscaling or the spray-drying was observed. Lipid droplet reduction after different doses of the extracts can also be observed in Figure 9. However, in this case, the longer exposure of cells to the extracts (9 days) also reduced the cell viability, especially during the first 2 days when the cells were still pre-adipocytes, making further studies necessary to increase the safety–activity balance of these extracts for anti-adipogenic purposes.

The application of subcritical water as a green protocol represents a crucial advance in metabolite extraction, as confirmed by the activity tests; therefore, after addressing the biological activity, further downstream processes were tested to increase the industrial applicability of the chestnut extracts. Notably, a critical point to address can be represented by the removal of water (i.e., product concentration) due to its high boiling point and the associated energy requirements. Accordingly, this study proposes the application of two possible solutions able to overcome this issue that are available at the industrial scale: membrane filtration and resin adsorption.

### 3.5. Membrane Filtration

A sample obtained by optimized MASWE (150 °C, 30 min, 1:30 S/L) was considered for membrane filtration (after clarification), by applying two different membrane mashes: those obtained by ultrafiltration (UF, 5000 Da) and those obtained by nanofiltration (NF, 150–300 Da). According to the different pore dimensions, the aim was to achieve metabolite fractionation (UF) or product concentration (NF). This approach is fundamental to remove the water and achieve the extraction, avoiding expensive work-up procedures that could also affect the product quality (i.e., evaporation and relative thermal degradation).

#### 3.5.1. Ultrafiltration (5000 Da)

The UF process was adopted to discriminate the polyphenolic fractions with high (tannins) and low molecular weights. TPC and antioxidant activity were used to describe the UF trend according to the retentate and permeate streams, by adopting the raw extract as a reference (Table 8). In addition, the flow rate of the process is reported in Figure S15.

**Figure 10:**
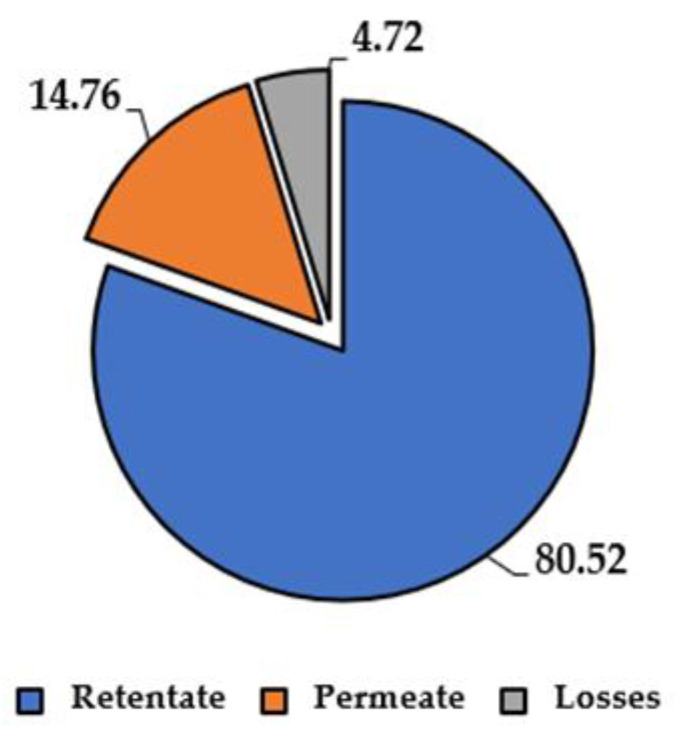
UF, TPC mass balance, expressed as percentage on TPC.

It is worth noting that with an average volume contraction of 63.56%, the retentate (heavier metabolites) led to a 16.61 mg/mL TPC, in contrast to the 2.16 mg/mL of the permeate. The overall TPC recovery (reflecting the process efficiency) was split into 80.52% vs. 14.76%, consolidating the assumption that tannins cover the majority of the extract composition (see Section 3.1.4). As reported by the pie chart in Figure 10, only 4.72% of losses were registered. In connection to the TPC variation, the antioxidant activity (expressed as IC_50_) does not show great fluctuations, indicating the presence of low molecular weight molecules that are extremely active. Shortly, in the retentate streams, it was possible to recover approx. 80% of the TPC in one-third of the water volume.

**Figure 11:**
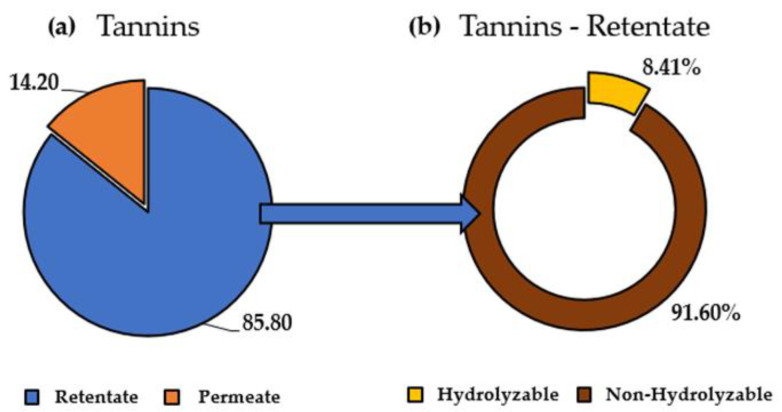
UF tannins mass balance, expressed as the percentage on TPC. (**a**) UF total tannin content; (**b**) tannin composition of retentate (hydrolyzable and nonhydrolyzable).

The cinchonine hemisulfate assay was applied to the UF retentate and permeate streams to quantify the recovery percentages with respect to the raw extract. As reported in Figure 11, 85.80% of the total tannins were contained in the retentate, as expected, of which 91.60% were nonhydrolyzable, roughly reflecting the original composition. Thus, the UF allowed us to concentrate the tannin-rich fraction in the retentate, fractionating from the low molecular weight contained in the permeate. It is worth noting that the entire UF protocol was performed in only 16 min, to process approx. 1 L of extract solution.

#### 3.5.2. Nanofiltration (150–300 Da)

The NF protocol exploited membranes with very narrow meshes, with the aim of concentrating the extract, reducing the metabolite dispersion at the minimum, and allowing the passage of water and salts in the permeate. The results reported in Table 8 and Table 9 show how, when facing a volume contraction comparable to that of UF (64.22% vs. 63.56%), the retentate contains most of the total amount of polyphenols (96.90% TPC recovery), bearing a concentration of 20.70 mg/mL against the 0.30 mg/mL permeate (with only 3.00% TPC recovery). Figure S16 shows the concentration trend during the NF process. In the latter NF streams, the cinchonine hemisulfate assay, as expected, showed a complete lack of tannins. It is worth noting that the entire NF protocol was performed in only 7 min, to process approx. 1 L of extract solution.

### 3.6. Resin Concentration

**Figure 12:**
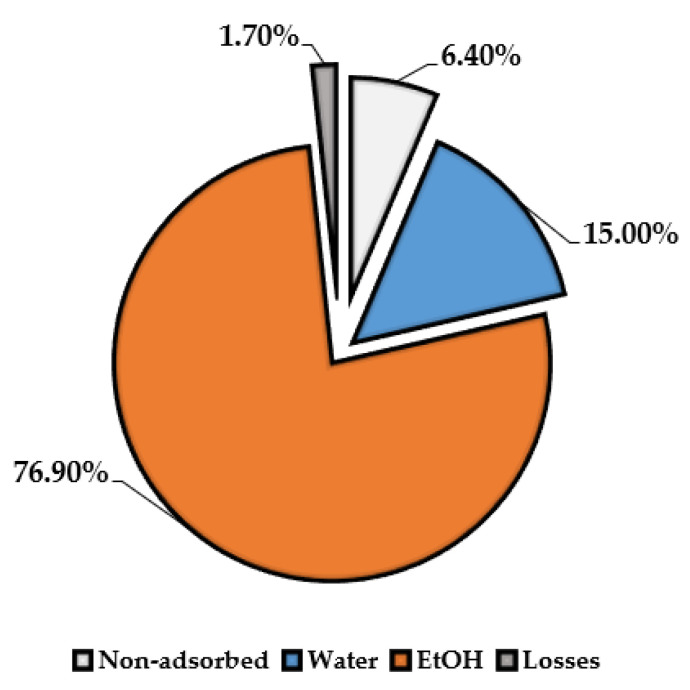
TPC recovery trend, depending on the different elution fractions.

As reported for the membrane filtration approach, it is possible to concentrate and purify the MASWE extract by means of an appropriate resin process. In this case, a macroporous resin (Sepabeads SP 825 L) was chosen in previous studies [9] to adsorb polyphenols selectively. The protocol was monitored by the TPC recovered for every fraction (raw extract was used as a reference), as reported in Figure 12. First, 6.40% of TPC recovery was detected in the “nonadsorbed fraction”, which was residual in the starting aqueous solution eluted through the resin bed. After that, the system was washed with 3 BV deionized water to remove the impurities and co-extracts, with the aim of concentrating the polyphenol composition of the final product. During this process, approx. 15.00% of the total TPC was removed from the system. Lastly, the adsorbed metabolites were removed from the macroporous resin with EtOH solution, which was able to recover 76.90% of the total TPC. The mass balance of the whole process shows a loss of 1.70% of TPC.

**Figure 13:**
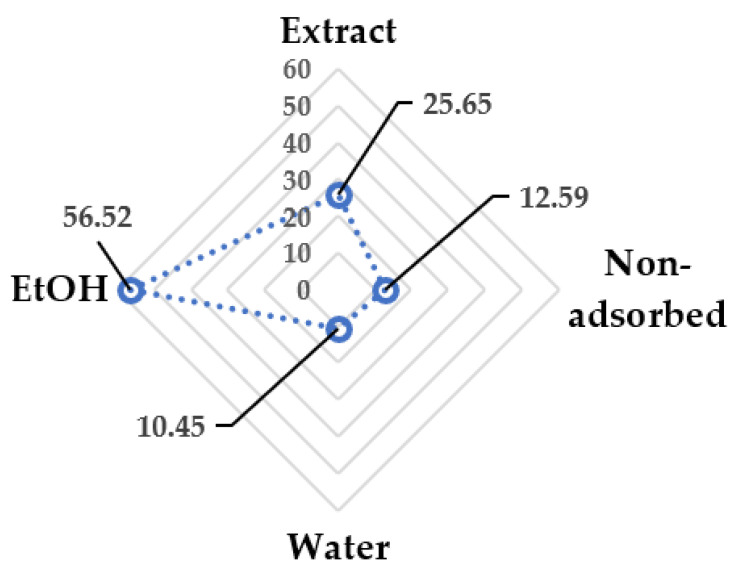
Purification trend of resin process. Reported values are expressed as percentages (mg_GAE_ on 100 mg_EXTR_).

The lower selectivity of “non-adsorbed” and “water” eluates (12.59% and 10.45%) supports the effective removal of undesired co-extracts (see Figure 13). These two fractions could be further processed to recover the remaining polyphenols, ideally with another resin purification. In summary, for the ethanolic fraction, the overall process allowed for the product to be concentrated at more than 30% (from 25.65% of the raw extract to 56.52%), recovering almost 77% of the total TPC. Furthermore, sampling across the EtOH elution allowed us to detect an extra-purified fraction, reaching 77.16% selectivity (mg_GAE_ on 100 mg_EXTR_) and representing a total TPC recovery of 37.0%.

## 4. Conclusions

Solvent-free extracts of chestnut peels have been obtained under SWE, first at the lab scale and then when scaled up to the semi-industrial level using a newly designed prototype. Comparison of SWE with recent work performed under conventional conditions may reveal a higher recovery of polyphenols in a much shorter extraction time. This technique not only allows significant process intensification but has also been shown to minimize metabolite degradation preserving the activity. It is worth noting that replacing ethanol or other organic solvents with water has remarkable economic, environmental, and safety benefits.

The recovered raw dry extracts (freeze-dried and spray-dried) have shown remarkable antioxidant power that has been confirmed in a biologically relevant model of adipocytes. Encouraging data on the adipocyte model showed the influence of chestnut extracts on adipocyte maturation and the consequent potential antiadipogenic activity that still requires further investigation to corroborate the claim. In perspective, the reported bioactivity of the extracts supports applications in functional foods or in nutraceutical products. The reduction of lipids absorption together with the relevant antioxidant and chelating activities show that the SWE of residual chestnut peels may provide a high-added-value product.

## Figures and Tables

**Table 1:** Temperature-related degradation: percent losses of yield and TPC for re-irradiated samples (from 150 °C to 220 °C).

	Degradation (%)		
S/L Ratio	Extraction Yield	TPC Yield	TPC Selectivity
1:20	72.54	75.17	9.80
1:30	70.48	74.64	14.09

**Table 2:** Time-related degradation: percent losses of yield and TPC for re-irradiated samples (from 30 min to 45 min).

	Degradation (%)		
S/L Ratio	Extraction Yield	TPC Yield	TPC Selectivity
1:20	18.39	42.32	23.71
1:30	17.24	39.68	27.08

**Table 3:** Kinetic parameters of the Peleg model. Determined for 1:20 and 1:30 extractions at 150 °C.

S/L Ratio	Key Parameter	B_0_	*C_max_*
		(mg_GAE_/g_DM_)/min	mg_GAE_/g_DM_
1:20	Extr. Yield	1666.67	294.12
	TPC Yield	15.50	9.54
1:30	Extr. Yield	555.56	384.62
	TPC Yield	6.13	14.12

**Table 4:** Main components of chestnut optimized extract under subcritical conditions.

Component	Rt	MW	mg/g_EXTR_	% (g/100g_EXTR_)
*Gallic acid*	5.45	170	0.90	0.09
*(Epi) catechin*	32.95	290	0.66	0.07
*Catechin-like comp.*	50.77	290	-	-
*Ellagic acid*	64.62	302	0.38	0.04
*(Epi) gallocatechin*	70.45	305	30.20	3.05
*Catechin*	74.80	290	112.85	11.28

**Table 5:** Total tannin quantification and composition (hydrolyzable and nonhydrolyzable) in SWE chestnut extract.

	mg/g_TPC_	% (g/100g_TPC_)
Total Tannins	828.40	82.84
Hydrolyzable	63.54	6.35 (*7.67 of total tannins*)
Nonhydrolyzable	764.86	76.49 (*92.33 of total tannins*)

Values are expressed as the total polyphenolic content (TPC).

**Table 6:** MASWE optimized extract characterization: antioxidant activity and Cu chelating activity (expressed as IC50 and Trolox^®^ eq. and EDTA eq., respectively).

	Antioxidant Activity		Cu chelating activity	
	IC50 (µg/mL)	Trolox^®^ eq. (mmol/g_EXTR_)	IC50 (mg/mL)	EDTA eq. (mmol/g_EXTR_)
MASWE extract	2.9	5.43	0.54	85.07

**Table 7:** Lab-scale and semi-industrial scale SWE comparison. Extraction yield, TPC, antioxidant, and chelating activity.

		TPC		Antioxidant Activity		Cu chelating Activity	
	Extraction Yield (mg_Extr_/g_DM_)	Selectivity (%, g_GAE_/g_Extr_)	Yield (mg_GAE_/g_DM_)	IC_50_ (µg/mL)	Trolox^®^ eq. (mmol/g_EXTR_)	IC_50_ (mg/mL)	EDTA eq. (mmol/g_EXTR_)
MASWE (Lab-scale)	416.56	38.96	160.70	2.9	5.43	0.54	85.07
Semi-industrial	394.20	30.38	119.74	4.2	3.75	0.88	51.72

**Table 8:** UF protocol. Volume contraction, TPC variations, antioxidant activity (DPPH), and mass balance of the process.

Sample	Volume Contraction	Concentration	TPC Recovery *	DPPH–IC50	Mass Balance
	(%)	(mg/mL)	(%)	(µg/mL)	(%)
Extract (*Ref*)	-	7.60	100	2.9	-
Retentate	63.56	16.61	80.52	5.3	98.05
Permeate	28.81	2.16	14.76	4.7	

* with respect to raw extract (Ref).

**Table 9:** NF protocol. Volume contraction, TPC variations, and mass balance of the process.

Sample	Volume Contraction	Concentration	TPC Recovery *	Mass Balance
	(%)	(mg/mL)	(%)	(%)
Extract (*Ref*)	-	7.60	-	-
Retentate	64.22	20.70	96.90	99.90
Permeate	35.78	0.30	3.00	

* with respect to raw extract (Ref).

## Data Availability

Data contained in the article.

## Supplementary Materials

The following supporting information can be downloaded at: https://www.mdpi.com/article/10.3390/antiox11050988/s1, Figure S1: LC-MS of chestnut peel extract, DAD chromatograms. (a) sample (obtained as b), re-irradiated at 220 °C; (b) extract at 150 °C, 30 min.; Figure S2: LC-MS of chestnut peel extract re-irradiated at 220 °C. ESI+ scan for pyrogallol detection [M+H]+:127.11 and relative DAD chromatogram. Figure S3: LC-MS of chestnut peel extract. ESI+ scan for gallic acid detection [M+H]+: 171 m/z.; Figure S4: LC-MS of chestnut peel extract. ESI+ scan for catechin detection [M+H]+: 291 m/z. Figure S5: LC-MS of chestnut peel extract. ESI+ scan for epicatechin detection [M+H]+: 291 m/z. Figure S6: LC-MS of chestnut peel extract. ESI+ scan for ellagic acid detection [M+H]+: 303 m/z. Figure S7: LC-MS of chestnut peel extract. ESI+ scan for (epi)gallocatechin detection [M+H]+: 306 m/z. Figure S8: ATR: Chestnut extract. Figure S9: ATR: Avicell^®^. Figure S10: ATR: Syloid^®^. Figure S11: ATR: Maltodextrin. Figure S12: ATR: Formulate chestnut extract/Avicell^®^. Figure S13: ATR: Formulate chestnut extract/Syloid^®^. Figure S14: ATR: Formulate chestnut extract/maltodextrin. Figure S15: Ultrafiltration of chestnut extract: process flow rate. Figure S16: Nanofiltration of chestnut extract: process concentration rate depending on permeate volume.
