# Supplementary material for: Bioactive Antioxidant Compounds from Chestnut Peels through Semi-Industrial Subcritical Water Extraction
Source: Antioxidants (Basel). 2022 May 18;11(5):988. doi: 10.3390/antiox11050988 (PMC9137501; doi:10.3390/antiox11050988)
Supplement: Supplementary file 1 [file antioxidants-11-00988-s001.zip › antioxidants-1720876-supplementary-done.pdf]

antioxidants

MDPI

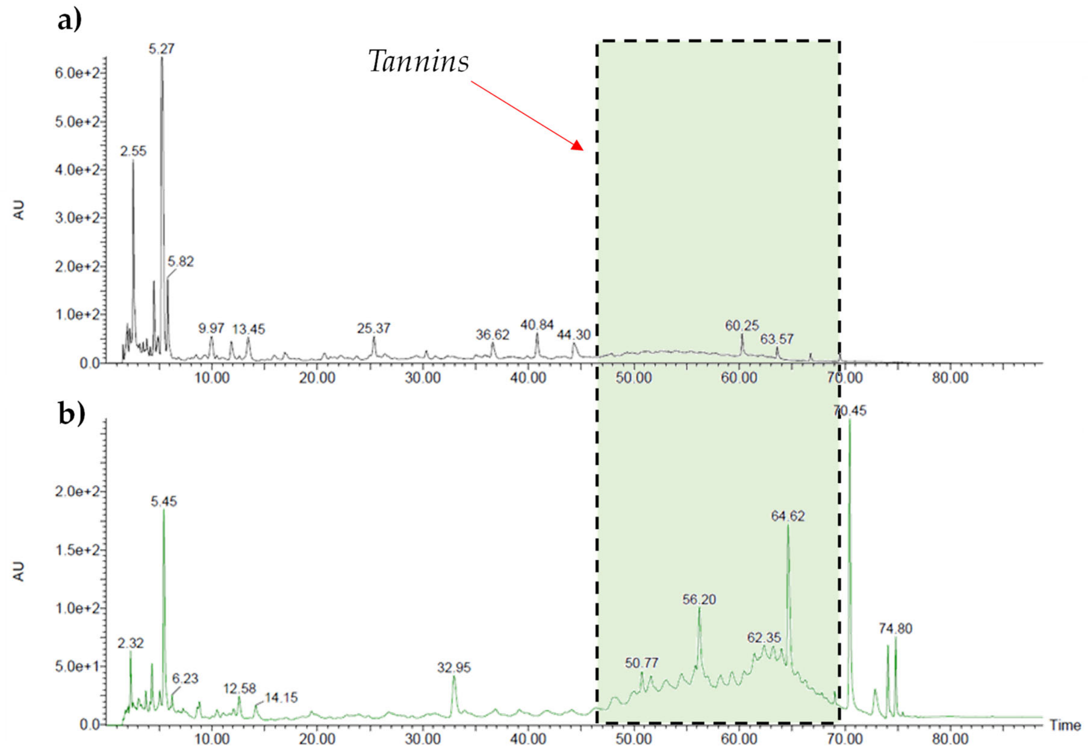

Figure S1. LC-MS of Chestnut peels extract, DAD chromatograms. a) sample (obtained as b), re-irradiated at 220 °C; b) extract at 150°C, 30 min.

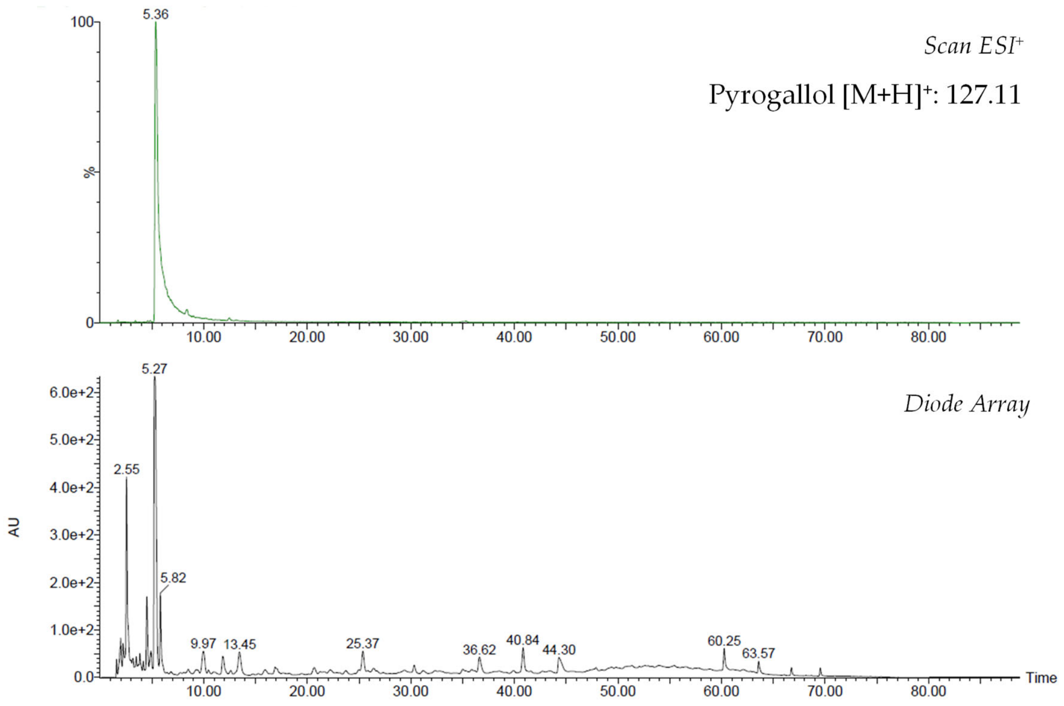

Figure S2. LC-MS of Chestnut peels extract re-irradiatd at 220 °C. Scan ESI+ for pyrogallo detection [M+H]+:127.11 and relative DAD chromatogram.

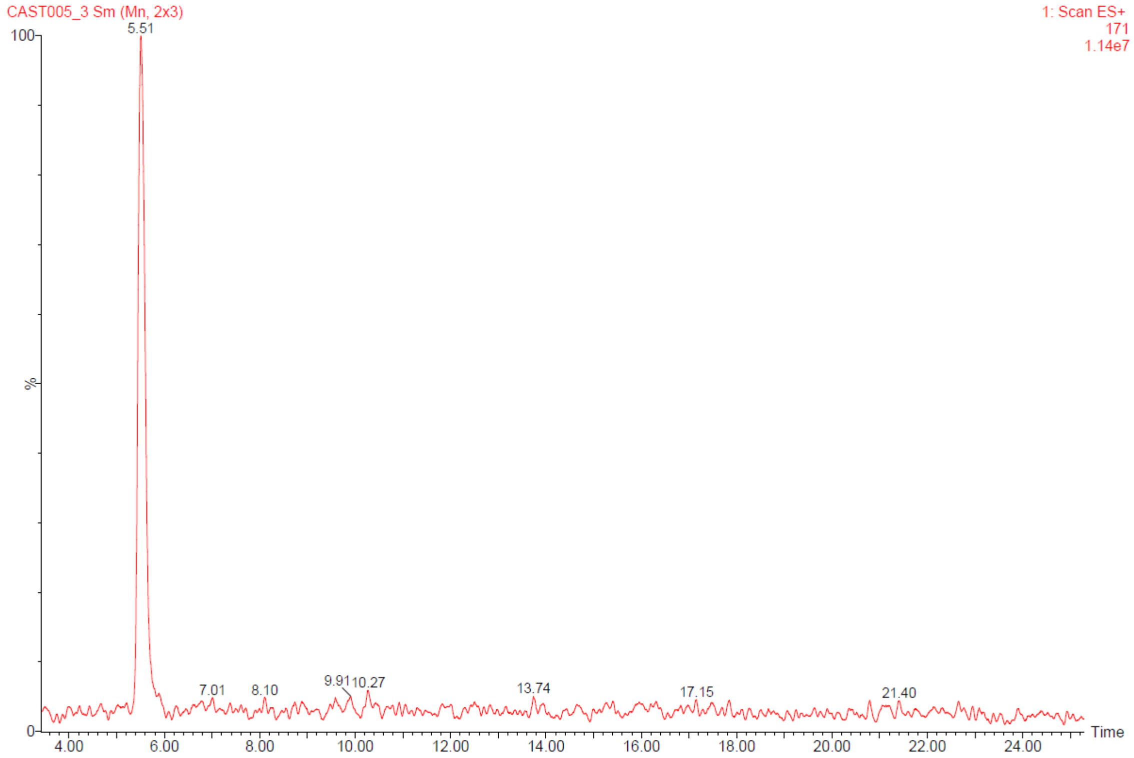

Figure S3. LC-MS of Chestnut peels extract. Scan ESI+ for gallic acid detection [M+H]+: 171 m/z.

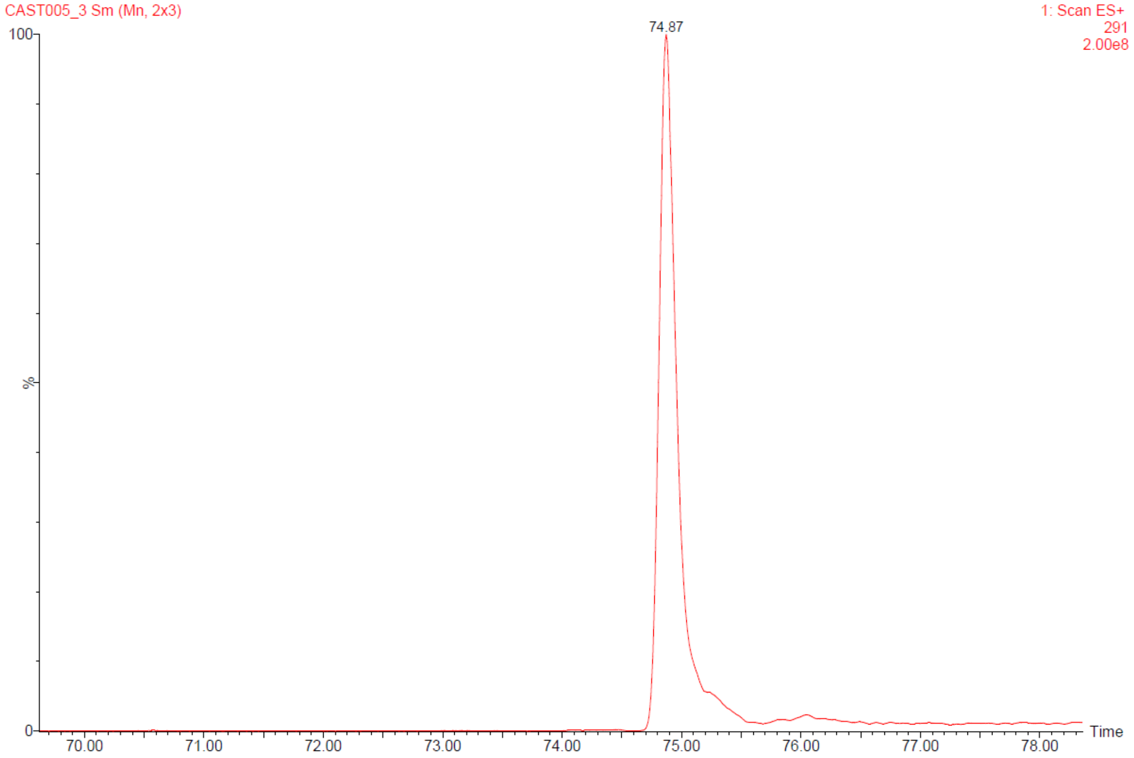

Figure S4. LC-MS of Chestnut peels extract. Scan ESI+ for catechin detection [M+H]+: 291 m/z.

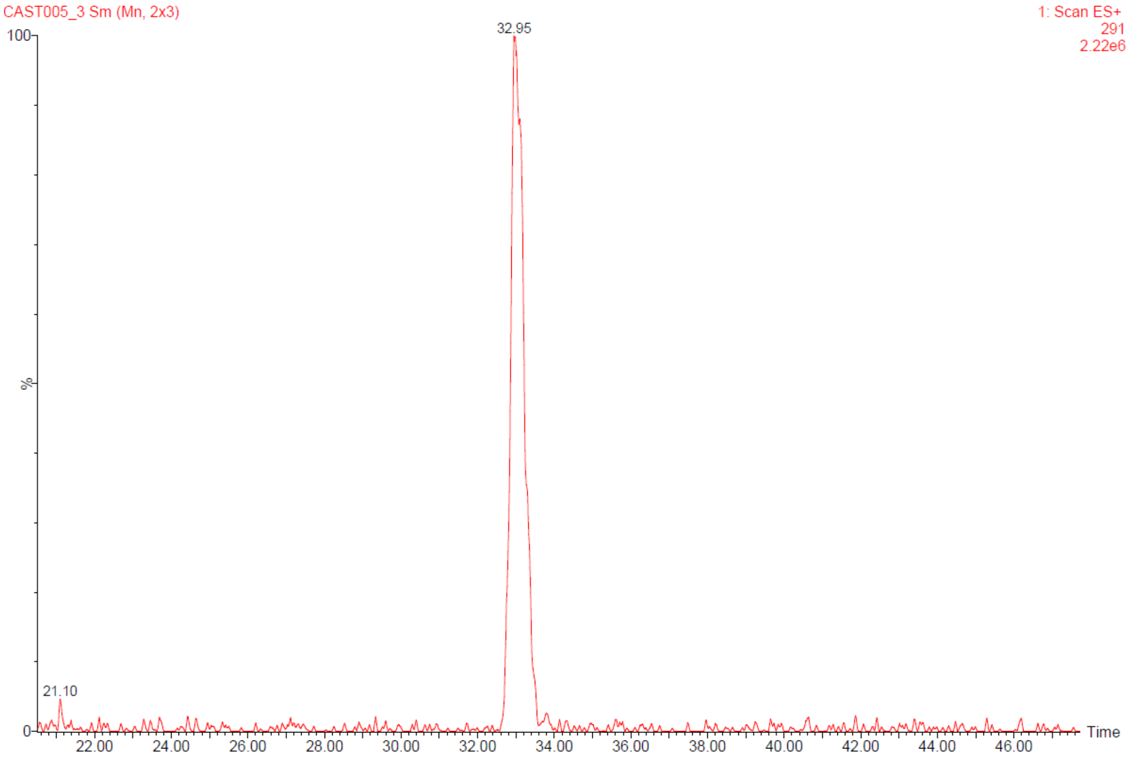

Figure S5. LC-MS of Chestnut peels extract. Scan ESI+ for epicatechin detection [M+H]+: 291 m/z.

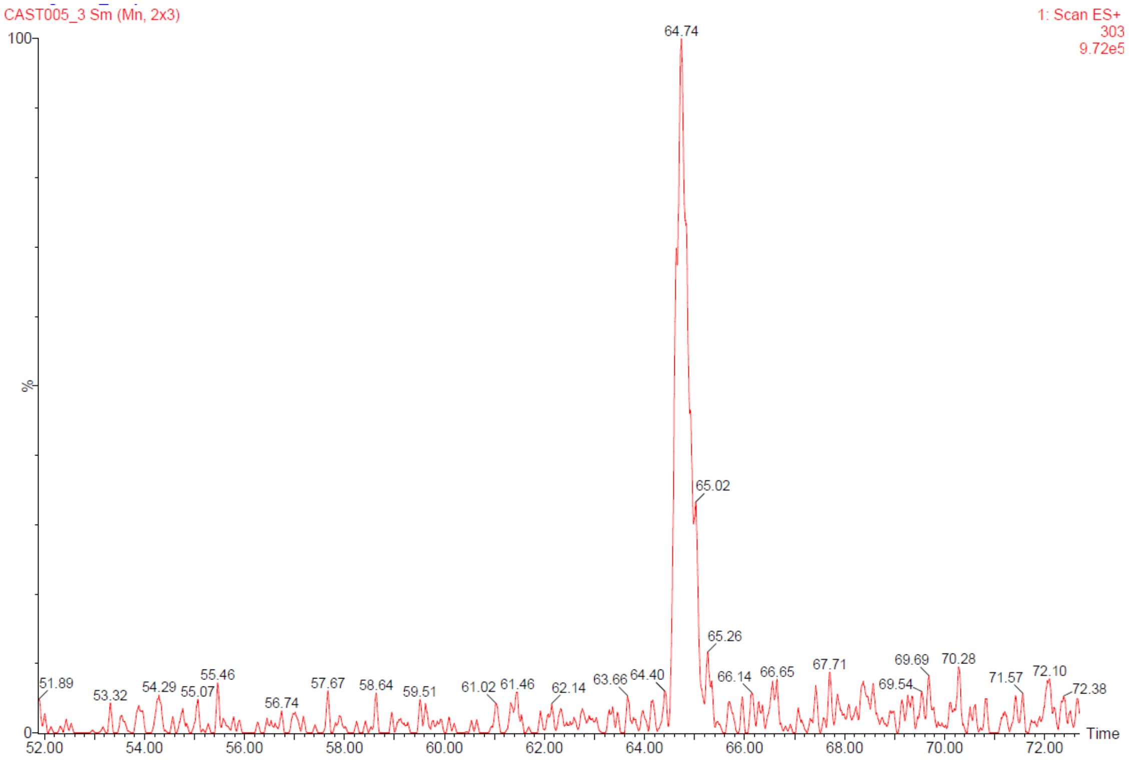

Figure S6. LC-MS of Chestnut peels extract. Scan ESI+ for ellagic acid detection [M+H]+: 303 m/z.

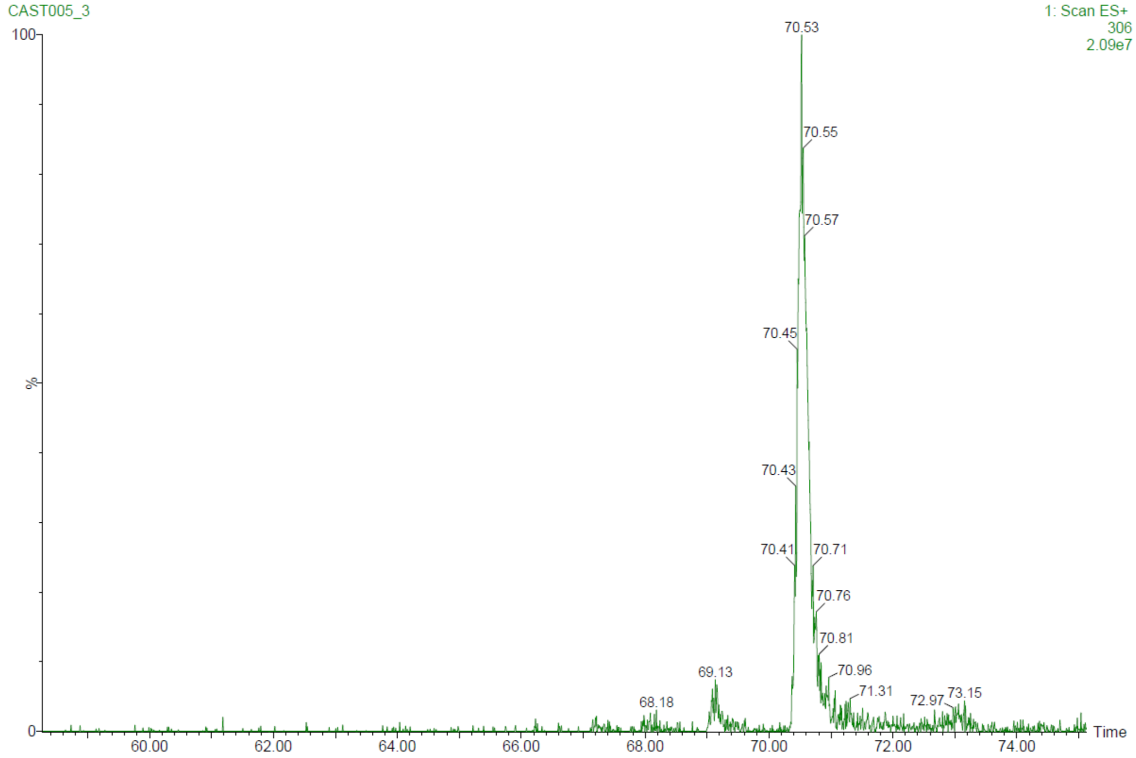

Figure S7. LC-MS of Chestnut peels extract. Scan ESI+ for (epi)gallo catechin detection [M+H]+: 306 m/z.

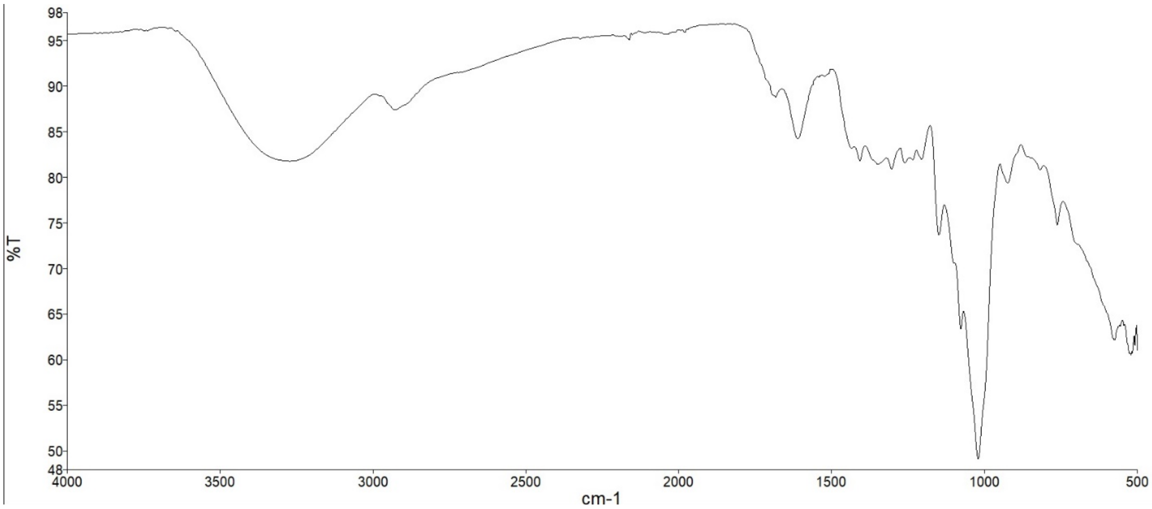

Figure S8. ATR: Chestnut extract.

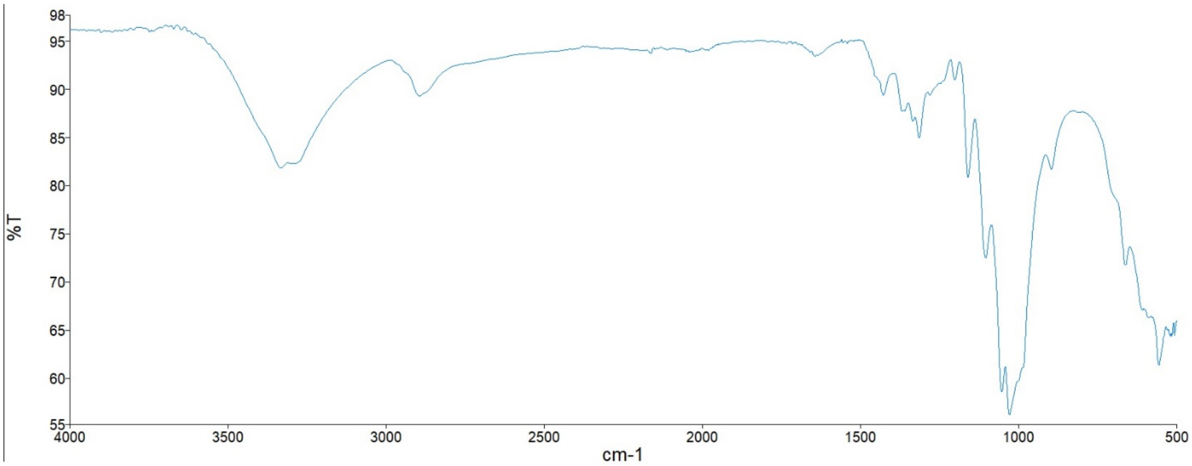

Figure S9. ATR: Avicell®.

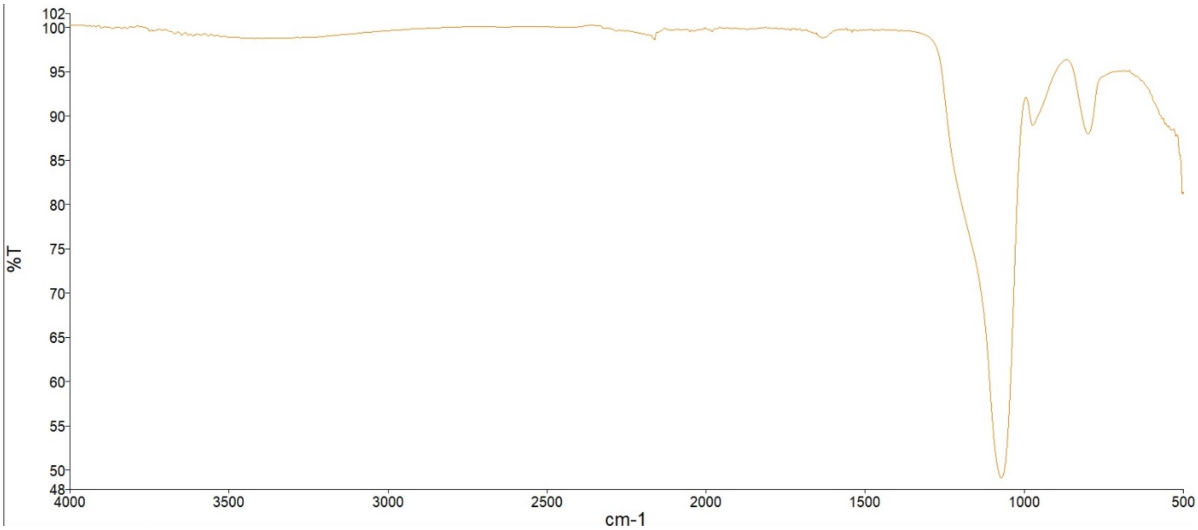

Figure S10. ATR: Syloid®.

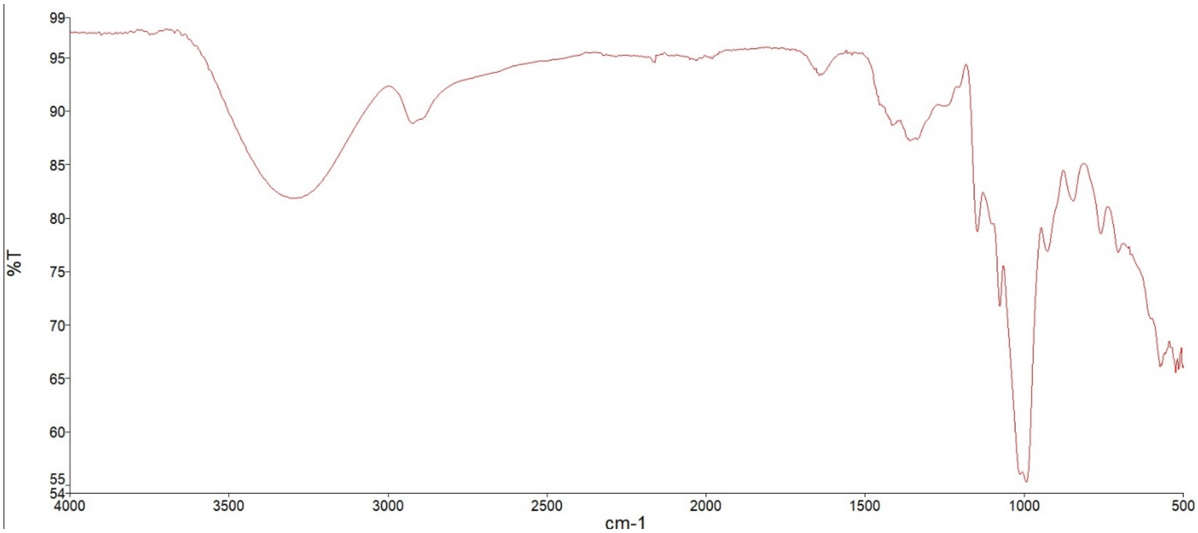

Figure S11. ATR: Maltodextrin.

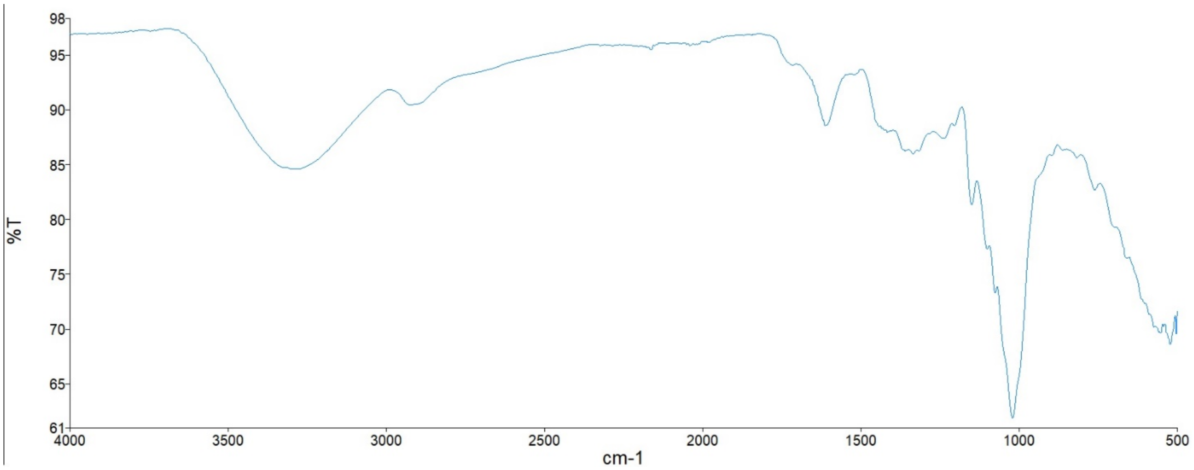

Figure S12. ATR: Formulate chestnut extract/Avicell®.

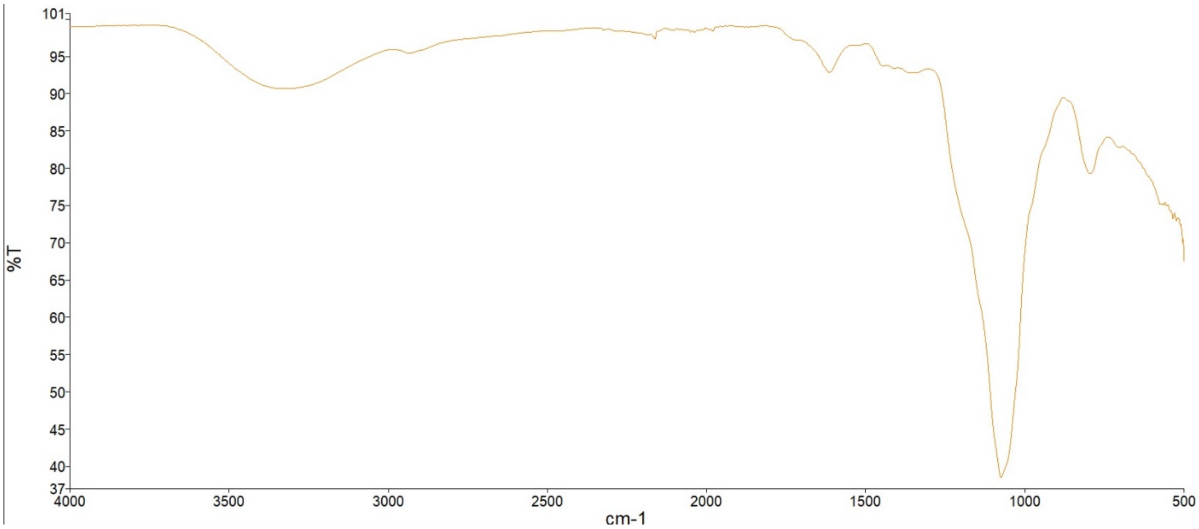

Figure S13. ATR: Formulate chestnut extract/Syloid®.

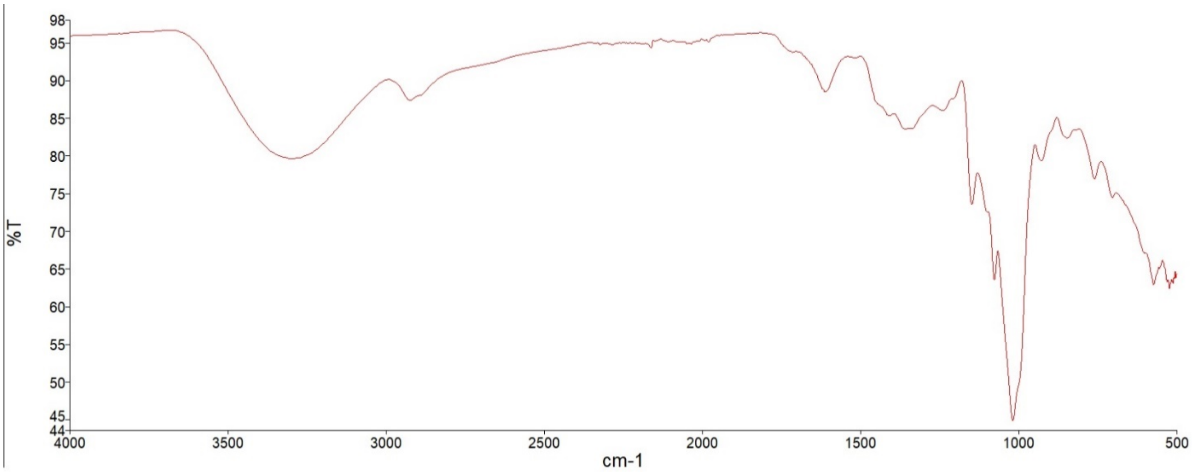

Figure S14. ATR: Formulate chestnut extract/Maltodextrin.

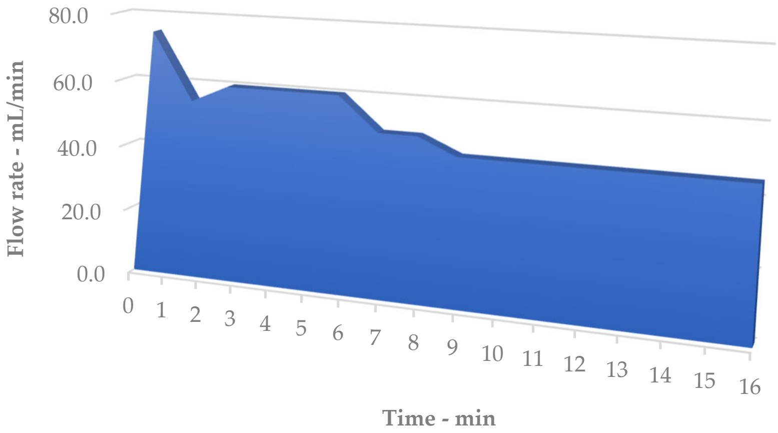

Figure S15. Ultrafiltration of Chestnut extract: process flow rate.

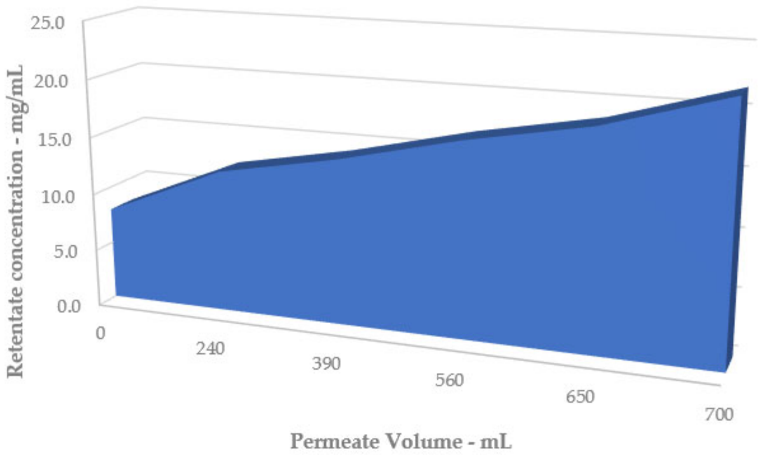

Figure S16. Nanofiltration of Chestnut extract: process concentration rate depending on permeate volume.
